# Genotyping of *Candidatus* Syngnamydia salmonis (*chlamydiales; Simkaniaceae*) co-cultured in *Paramoeba perurans* (amoebozoa; Paramoebidae)

**DOI:** 10.1007/s00203-018-1488-0

**Published:** 2018-02-17

**Authors:** Are Nylund, Dario Pistone, Christiane Trösse, Steffen Blindheim, Linda Andersen, Heidrun Plarre

**Affiliations:** 1 Department of Biology, University of Bergen, Thormohlensgt 55, 5020 Bergen, Norway; 2 The Industrial and Aquatic Laboratory (ILAB), Thormohlensgt 55, 5020 Bergen, Norway

**Keywords:** *Chlamydiales*, *Simkaniaceae*, *Syngnamydia salm*onis, *Paramoeba perurans*

## Abstract

*Candidatus* Syngnamydia salmonis (*Chlamydiales, Simkaniaceae*) was described as an epitheliocystis-causing bacterium from the gills of Atlantic salmon (*Salmo salar*) in Norway. A bacterium showing 99.2% 16S rRNA identity to *Cand*. S. salmonis is able to multiply in *Paramoeba perurans* and based on the classification criteria this bacterium could represent the same species as *Cand*. S. salmonis. Sequencing the genome of the cultured bacterium has made it possible to fulfill the minimal standards for genetic characterization of species within the order Chlamydiales. The complete rRNA genes, the amino acid sequences of SucA, PepF, Adk, HemL, DnaA, FtsK and FabI, are presented in addition to the morphology of the Chlamydia-like morphs in the cytoplasm of *P. perurans*.

## Introduction

The phylum *Chlamydiae* consists of intracellular bacteria that show a biphasic developmental cycle in eukaryotic cells (Bedson and Bland 1932; Abdelrahman and Belland 2005; Horn 2008). They infect a wide range of different hosts including vertebrates, arthropods and amoeba, where they can be pathogens or endosymbionts. The chlamydial taxonomy has experienced numerous changes throughout the decades, but the accumulation of molecular data has made it clear that the phylum consists of several species belonging to different families; *Chlamydiaceae, Simkaniaceae, Waddliaceae, Parachlamydiaceae, Rhabdochlamydiaceae, Criblamydiaceae*, and four *Candidatus* families (Piscichlamydiaceae, Clavichlamydiaceae, Actinochlamydiaceae, and Parilichlamydiaceae) (Drahgi et al. 2004; Horn 2008; Karlsen et al. 2008; Steigen et al. 2013; Stride et al. 2013; Pawlikowska-Warych and Deptula 2016). Bacterial taxonomy is based on a polyphasic approach that combines phenotypic and genetic characteristics. However, unlike other orders of bacteria there are a high number of *Candidatus* species described as members of *Chlamydiales* due to the lack of proper culture systems. The *Chlamydiae* exhibit few morphological traits and the taxonomic affiliation is mainly based on phylogenetic analysis of 16S rRNA and housekeeping genes (Greub 2010, 2013). Recently, it was recommended that nine protein sequences should be used, in addition to the rRNA genes, to precisely classify newly discovered members of *Chlamydiales* at the family, genus and species levels (Pillonel et al. 2015).

Members of the four candidatus families have all been obtained and detected intracellularly, mainly, in gill epithelial cells in different fish species (Drahgi et al. 2004; Karlsen et al. 2008; Steigen et al. 2013; Stride et al. 2013). While members of the different families in *Chlamydiales*, with the exception of *Chlamydiaceae*, are all cultured in free living amoebae which are believed to be natural reservoirs for these chlamydia-like organisms (CLO) in the different environments, it has not been possible to culture any of the fish CLOs in artificial media or cell cultures. However, during isolation of the causative agent of amoebic gill disease (AGD), *Paramoeba perurans*, from Atlantic salmon (*Salmo salar*) it was discovered that some of the cultures were positive for *Candidatus* Syngnamydia salmonis (family *Simkaniaceae*). This bacterium has previously been described as an epitheliocystis causing agent on the gills of Atlantic salmon (Nylund et al. 2015). Co-culturing *Cand*. S. salmonis in clonal cultures of *P. perurans* led to an increase in the amount of both, showing that the bacterium is able to multiply in the amoeba.

In the present study, *Cand*. Syngnamydia salmonis is co-cultured in *P. perurans* and genetically characterized according to the recommended taxonomical approach given by Greub (2013) and Pillonel et al. (2015). Its developmental stages in *P. perurans* are described and analysis of rRNA genes and a selection of housekeeping genes are presented and discussed.

## Materials and methods

### Isolation and culturing of *Paramoeba perurans*

Isolates of *P. perurans* were obtained from gills of farmed Atlantic salmon (*Salmo salar*) collected in western Norway. The primary isolates were cultured on a Malt-Yeast Agar (MYA; 0.01% Bacto™ Malt Extract, 0.01% Bacto™ Yeast Extract, 2.0 Bacto™ Agar in 34‰ sterile sea water) with a layer of sterile sea water covering the gill tissue (Crosbie et al. 2012). The cultures were incubated at 15 °C and the amoebas were passed through three passages before cloning. To establish clonal lines from cultures of *Paramoeba perurans* isolates, single floating trophozoites were pipetted into separate wells of 24-well cell culture plates and 500 µl malt yeast broth (MYB; 0.01% Bacto™ Malt Extract, 0.01% Bacto™ Yeast Extract in 34‰ sterile sea water) were added to each well. Dividing cells were monitored and passaged to larger culture vessels after reaching the appropriate density of floating trophozoites. The species, *Paramoeba perurans*, was confirmed by sequencing of the partial small subunit rRNA gene. The clonal cultures of *P. perurans* are feeding on live bacteria that followed the amoeba during isolation from the salmon gills. The dominating bacteria belong to phylum *Proteobacteria* (about 95.0%) and the majority of these are members of family *Vibrionaceae* (about 88.0%). It has not been possible to culture *P. perurans* in media containing dead bacteria only. Based on real time RT PCR, a majority of the isolates of *P. perurans* were also positive for the bacterium *Candidatus* Syngnamydia salmonis (Nylund et al. 2015), and clones from two of these isolates (H03/14Pp and R18/15Pp) were sent for Illumina sequencing of the genome.

### Illumina sequencing

Total DNA and RNA were isolated from clonal cultures of *Paramoeba perurans* (isolates H03/14Pp and R18/15Pp) that were strongly positive, by real time RT PCR, for the presence of *Candidatus* Syngnamydia salmonis. The DNA and RNA were sent to BaseClear (BaseClear Group, Netherlands) for Illumina (Illumina Casava pipeline version 1.8.3) sequencing. Quality-filtered sequence reads were puzzled into a number of contig sequences. The assembly was performed using the “*De novo* assembly” option of the CLC Genomics Workbench version 7.0 (CLCbio). The contigs were linked and placed into scaffolds or supercontigs using the SSPACE Premium scaffolder version 2.3 (Boetzer et al. 2011). BLAST analysis of the scaffolds was performed with the blastn program of ncbi-blast + version 2.2.29 (Camacho et al. 2009) using an e-value cutoff of 0.01. Based on taxonomic lineage of each scaffold, the scaffold sequences were divided into different categories: Amoebozoa (taxid 554,915), Euglenozoa (taxid: 33,682), Chlamydiae (taxid: 204,428), Bacteria (taxid: 2), and no match with the abovementioned categories. The results of *De Novo* assembly are presented in Table 1. The contigs, obtained from the Illumina sequencing of the two *P. perurans* clones, contained about 2 million nucleotides from a putative member of family *Simkaniaceae*.

**Table 1:** De Novo assembly statistics for Illumina sequencing of DNA and RNA from the *P. perurans* clone H03/14Pp

	Amoebozoa	Euglenozoa	Chlamydiae	Bacteria	Other
DNA					
No scaffolds	19	38	17	4182	13,201
Sum (bp)	280,203	234 169	2 155 523	56,480,364	13,438,851
Average size	1484	6162	126,795	13,505	1018
RNA					
No scaffolds	59	39	63	4300	23,555
Sum (bp)	107,572	195,540	196,933	11,460,084	31,487,174
Average size	1823	5013	3125	2665	1336

### Co-culturing of *Candidatus* Syngnamydia salmonis and *P. perurans*

*Cand*. S. salmonis was co-cultured with different clones of *P. perurans*. Growth of the bacterium was assessed using real time RT PCR assays targeting the amoebae, the bacterium and the added spike, Salmonid alphavirus, SAV (assay Nsp1, Andersen et al. 2007). The co-cultures were kept for 8 days at 15 °C and samples (triplicates) were taken after 2 h, 1, 2, 3, 4, and 8 days. The samples were added salmon alphavirus (SAV3) before extraction of RNA. A culture of *P. perurans* (H02/13Pp) lacking *Cand*. S. salmonis was kept as a control for the growth rates of the infected clones (H03/14Pp and R18/15Pp) and as a negative control for the *Cand*. S. salmonis real time RT PCR assay (Sch).

### Real time RT PCR

A new, specific, real time RT PCR assay was developed targeting the 18S rRNA of *Paramoeba perurans* (Assay: Pperu-F: GAT AAC CGT GGT AAA TCT AGA GCT AAT A. Pperu-probe: CTG GTT CTT TCG RGA GC. Pperu-R: TGG CAT TGG CTT TTG AAT CT. Efficiency = 1.97), while an assay targeting the 16S rRNA from *Cand*. S. salmonis (Assay: Sch, efficiency = 2.04) has already been published (Nylund et al. 2015). Standard curves for the two assays were generated using a tenfold dilution series of RNA in triplicate. The PCR efficiency (*E* = 10^1/(-*s*)^ − 1), regression analysis and standard curve slopes (Ct-value vs. log quantity) of the various assays were calculated from the obtained Ct-values. Salmonid alphaviruses (SAV3) were added prior to RNA extraction from the co-cultures of *P. perurans* and *Cand*. S. salmonis and used as an exogenous control. A previously developed assay (nsP1, efficiency *E* = 1.94), Andersen et al. (2007), targeting SAV was used as a standard to calculate the mean normalized expression (MNE) of 16S and 18S rRNA from *Cand*. S. salmonis and *P. perurans* during the culture period. Samples from all time points after the start of the co-cultures were taken as triplicates.

### Histology and TEM

Cultures of *P. perurans* were fixed in a modified Karnovsky fixative. The cultures were processed and sectioned as described in Steigen et al. (2013), and ultrathin sections were used for transmission electron microscopy (TEM) for detection of chlamydia-like bacteria in the amoeba, *P. perurans*.

### Identification of genes from *Cand*. S. salmonis

Blast search (blastx, NCBI), using contigs from the Illumina sequencing, was used for identification of genes coding for the proteins recommended by Pillonel et al. (2015) for precise genotyping of chlamydia-like organisms at the family, genus and species level. The putative amino acid sequences showed the highest similarity to *Simkaniaceae negevensis* and the sequence identity of the proteins were compared with those of *S. negevensis* Z (Collingro et al. 2011).

Partial rRNA genes (Accession no: KT158462) from *Candidatus* S. salmonis were also obtained directly from the gills of Atlantic salmon infected with *P. perurans*. The salmon was farmed in Hordaland County, Norway.

### Phylogeny

The phylogenetic analyses were performed for the 16S rRNA gene sequences of *Candidatus* Syngnamydia salmonis from two different cultures (H03/14Pp and R18/15Pp) of *P. perurans* and the corresponding gene sequences available in the GenBank. Sequence alignments were performed using Vector NTI and manual edited in Gene Doc. The phylogenetic tree was calculated in Tree Puzzle using the maximum-likelihood (ML) method available in TREE_PUZZLE 5.2 (available at: http://www.tree-puzzle.de), employing the HKY model of nucleotide substitution (Hasegawa et al. 1985). The phylogenetic tree was drawn in Tree View (Page 1996).

## Results

### Culture and morphology of *Cand*. S. salmonis

**Fig. 1:**
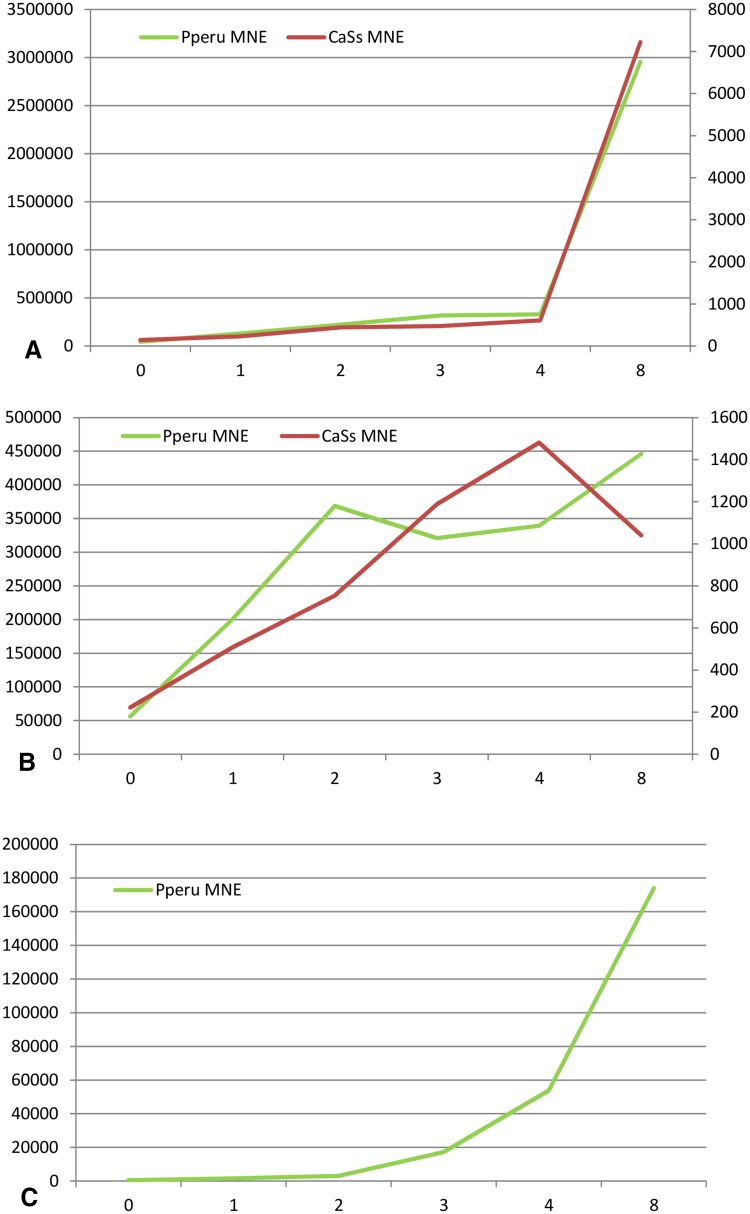
Growth of *Candidatus* Syngnamydia salmonis in co-culture with *Paramoeba perurans*; **A** Isolate H03/14Pp and **B** Isolate R18/15Pp. **C** Growth of isolate H02/13Pp without presence of *Candidatus* S. salmonis. MNE of *Paramoeba perurans* (left *y-*axis) in culture during a period of 8 days. MNE of *Candidatus* S. salmonis (right *y-*axis) in co-culture with *P. perurans* during a period of 8 days

*Paramoeba perurans* was co-cultured with *Candidatus* Syngnamydia salmonis (Fig. 1a, b) but the cultures did not seem to be negatively affected by the presence of the bacterium. Mean normalized expression (MNE) of the 18S rRNA from *P. perurans* reached a peak between 4 and 8 days at 15 °C coinciding with a peak in MNE of the 16S rRNA from *Cand*. S. salmonis. The *P. perurans* clone, H02/13Pp, was cultured without the presence of *Cand.* S. salmonis and the peak in the expression of 18S rRNA was reached after 8 days (Fig. 1c). The majority of isolates of this amoeba obtained from farmed Atlantic salmon (*Salmo salar*) suffering from amoebic gill disease are positive for the presence of this *Candidatus* species. However, *P. perurans* isolates may also be negative for the presence of *Cand*. S. salmonis and gills of the salmon can be positive for the bacterium without any presence of the amoeba, which suggest that *Cand*. S. salmonis is not an obligate symbiont of this amoeba species.

**Fig. 2:**
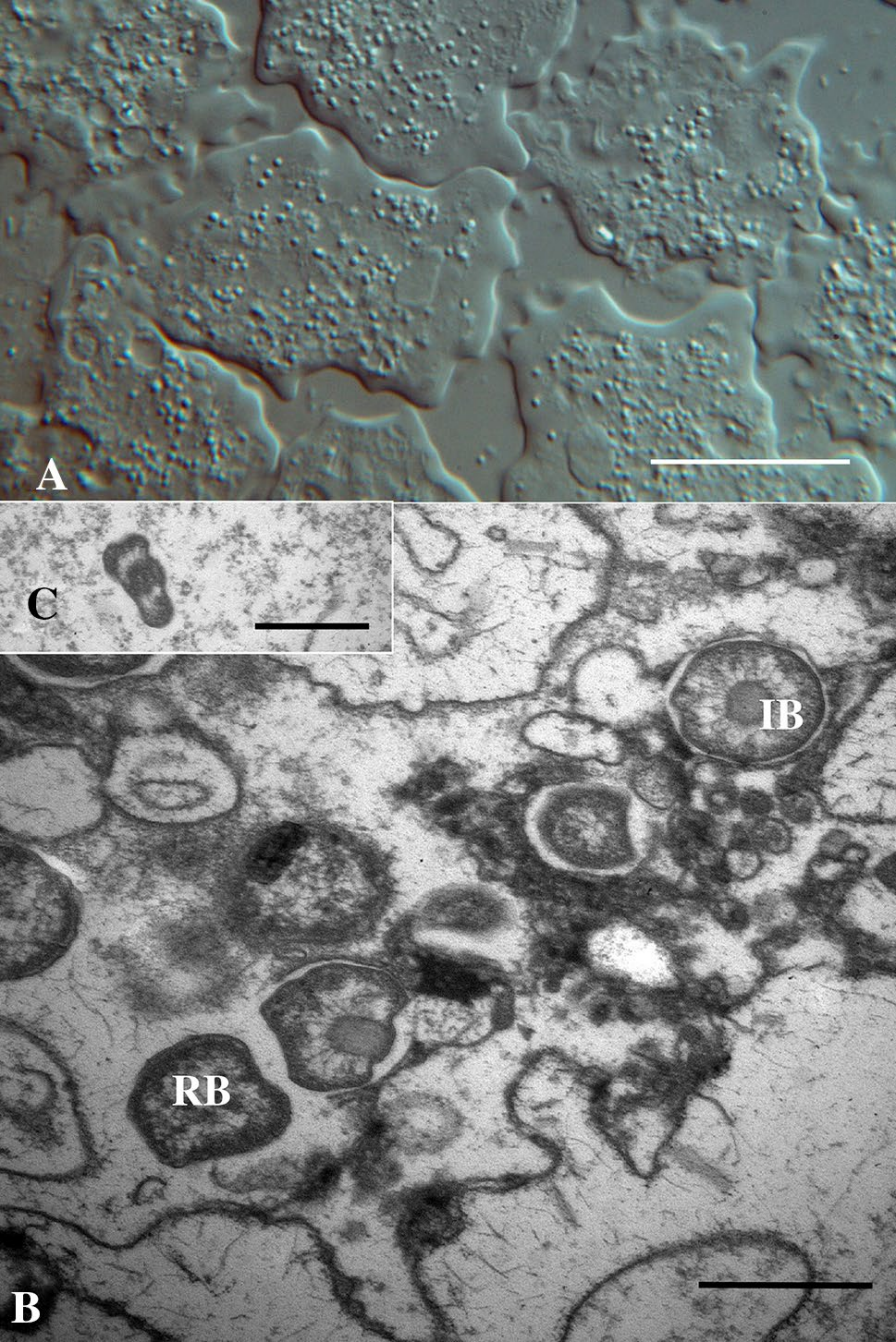
**A** Clonal culture of *Paramoeba perurans* (Isolate H03/14Pp). Bar = 25 µm. **B** *P. perurans* with chlamydia-like bacteria in the cytoplasm. Reticulate bodies (RB) and intermediate bodies (IB). Bar = 500 nm. **C** Elementary body. Bar = 500 nm

The morphology of the chlamydia-like bacteria in infected *P. perurans*, clone H03/14Pp (Fig. 2a), was obtained by the use of transmission electron microscopy (TEM). Ultrathin sections showed the presence of putative reticulate body (RB), intermediate body (IB) and elementary body (EB)—like morphs (Fig. 2b, c). All three morphs were seen free in the cytoplasm in most of the amoeba in infected cultures, while inclusions as seen in the epithelial cells of Atlantic salmon suffering from epitheliocystis, were not seen in the infected amoeba. Dividing RB-like morphs were seen in most amoebae and the largest diameter of the spherical RBs and IBs were 550 and 500 nm, respectively. The largest diameter of the flattened, oval, EBs was 400 nm, while the shortest diameter was 197 nm (Fig. 2c).

### Genotyping of *Cand*. S. salmonis

**Fig. 3:**
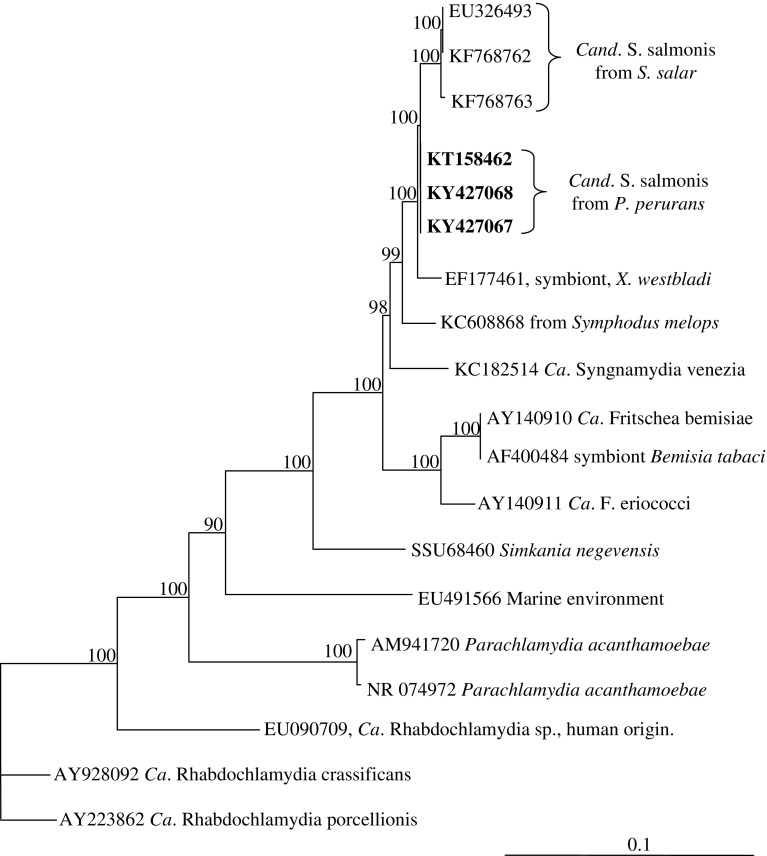
Phylogenetic tree showing the relationship between *Candidatus* Syngnamydia salmonis from *Paramoeba perurans* and Atlantic salmon *(Salmo salar)* and selected members of the family *Simkaniaceae*. The analysis is based on a 1342 nt long edited alignment of 16S rDNA sequences. Members of the families *Rhabdochlamydiaceae* and *Parachlamydiaceae* have been used as outgroup. The scale bar shows the number of nucleotide substitutions as a proportion of branch lengths

A partial genome of *Cand*. S. salmonis, consisting of 2.16 Mbp divided into 15 scaffolds (ranging from 31,207 to 531,909 bp), was obtained from the isolate H03/14CaSs in the *P. perurans* clone (H03/14Pp), and a partial genome of a similar size, divided into 50 contigs, was also obtained from isolate R18/15CaSs (in *P. perurans* clone R18/15Pp). A selection of open reading frames (ORFs) coding for putative protein sequences in addition to the complete rRNA genes were selected from the two partial genomes. These nucleotide sequences and putative amino acid sequences were used for a comparison of *Cand*. S. salmonis co-cultured in *P. perurans* with that of other members of *Chlamydiales* and family *Simkaniaceae*. The phylogenetic position of *Cand*. S. salmonis constructed from analysis of the 16S rRNA gene available in the GenBank cluster this *Candidatus* species in the family *Simkaniaceae* (Fig. 3). *Cand*. S. salmonis represents a distinct species within the family *Simkaniaceae* with the symbiont of *Xenoturbella westbladi* as the closest relative. A chlamydia-like 16S sequence (Accession no: KC608868) obtained from the gills of the wrasse, *Symphodus melops* (Steigen et al. 2015), also groups close to *Cand*. S. salmonis cultured in *P. perurans*. This wrasse specimen was negative for the presence of *Paramoeba* spp. on the gills (A. Nylund, Pers. Obs.).

A representative set of *Chlamydiae* housekeeping genes (amino acid sequences), DnaA, SucA, FabI, FtsK, PepF, Adk and HemL, recommended in the classification scheme suggested by Pillonel et al. (2015) are presented in Table 3. Following this scheme the 16S sequence shows 93.3% similarity to *S. negevensis*, while the 23S show an identity of 88.1%. *Cand*. S. salmonis lack the group I intron found in the I-CpaI target site of the 23S rRNA gene of *Simkania negevensis* (Table 2). The amino acid sequences of DnaA, SucA and FabI showed an identity to *S. negevensis* of 71.8, 64.6 and 78.8%, respectively, while the putative amino acid sequences identities of FtsK, PepF, Adk, and HemL compared to *S*. *negevensis* were 71.1, 63.6, 69.3, and 62.4%, respectively (Table 3).

**Table 2:** Comparison of rRNA genes from *Candidatus* Syngnamydia salmonis (Accession nos: KY427067, KY427068) cultured in *Paramoeba perurans* with *Candidatus* Syngnamydia salmonis obtained from the gills of *Salmo salar, Candidatus* Syngnamydia venezia from *Syngnathus typhle, Simkania negevensis, Candidatus* Fritschea bemisiae, *Candidatus* F. eriococci, and the chlamydial symbiont of *Xenoturbella*. A chlamydia sequence obtained from the gills of the wrasse, *Symphodus melops*, is also included (Accession no: KC608868)

Species	16S	ITS	23S	Accession no
*Cand*. S. salmonis	1538nt	365nt	3033nt	KY427067/68
*Cand*. S. salmonis	1349–99.2%	–	731–99.7%	KF768762
SM081012-5	1506–97.7%	386–80.5%	394–94.2%	KC608868
*Cand*. S. venezia	1477–96.4%	–	–	KC182514
Symbiont *Xenoturbella*	1530–98.8%	365–94.8%	2873–93.9%	EF177461
*Cand*. F. eriococci	1514–95.0%	400–73.6%	2615–89.4%	AY140911
*Cand*. F. bemisiae	1538–94.7%	393–68.8%	3054–89.2%	AY140910
*S. negevensis*	1545–93.3%	292–55.1%	3045–88.1%	U68460

**Table 3:** Amino acid sequence identity of selected proteins from *Simkania negevensis* (accession no: FR872582) and *Candidatus* Syngnamydia salmonis from the *P. perurans* clones H03/14Pp and R18/15Pp (Accession no)

Protein		Accession no	*Cand*. S. salmonis	*S. negevensis*	Amino acid
			No. aa	No. aa	identity (%)
2-Oxyglutarate dehydrogenase subunit E1	SucA	MG017632/33	900	909	64.6
Chromosomal replication initiation protein	DnaA	MG017622/23	444	445	71.8
Enoyl-ACP reductase	FabI	MG017624/25	307	309	78.8
Oligoendopeptidase F	PepF	MG017630/31	604	603	63.6
Adenylate kinase	Adk	MG017618/19	205	204	69.3
Glutamate-1-semialdehyde aminotransferase	HemL	MG017628/29	433	433	62.4
Cell division protein	FtsK	MG017626/27	750	761	71.1
Tyrosyl-tRNA synthetase	TyrS	MG017634/35	421	424	68.2
TLC ATP/ADP transporter	Atp/Adp	MG017620/21	529	528	66.5

## Discussion

A set of requirements, a minimal standard, for description of new species within *Chlamydiales* have been suggested by the Subcommittee on the taxonomy of *Chlamydiae* (Greub 2013). This standard states that a single strain cultured in any cell culture system including amoebal co-culture is sufficient for the description of a new species. In the present study, a strain of *Candidatus* Syngnamydia salmonis has been cultured in a clonal culture of *Paramoeba perurans* thus fulfilling this requirement of the minimal standard. However, the amoeba, *P. perurans*, and the *Cand*. Syngnamydia salmonis have not been deposited in two independent culture collections which is also part of the minimal requirement for description of new species. Thus, all the requirements for description of new species within the order Chlamydiales have yet to be fulfilled.

The chlamydia-like bacterial morphs seen in the *P. perurans* clones, infected with *Cand*. S. salmonis, were not surrounded by an inclusion membrane, while all morphs of this bacterium resided in large inclusions in infected epithelial cells from salmon (Nylund et al. 2015). *Simkania negevensis* has only been found within inclusion membranes in the amoeba host *Acanthamoeba polyphaga* (Kahane et al. 2001). However, the lack of inclusion membranes has also been described from the amoebas *Vermamoeba vermiformis* and *Hartmannella vermiformis* infected with *Rubidus massiliensis, Neochlamydia hartmannellae, Protochlamydia massiliensis, Protochlamydia phocaeensis*, and *Waddlia chondrophila*, respectively (Horn et al. 2000; Michel et al. 2004; Khalil et al. 2016, 2017; Benamar et al. 2017). *P. massiliensis* and *P. phocaeensis* were both able to form inclusion vacuoles in *Acanthamoeba castellani* (Benamar et al. 2017; Khalil et al. 2017). It has been speculated that the lack of an inclusion membrane is a result of host cell response (Benamar et al. 2017).

The morphology and the 16S rRNA sequence of *Cand*. S. salmonis have already been published as one of several bacteria causing epitheliocystis in Atlantic salmon (*Salmo salar*) (Nylund et al. 2015). DIG-labelled RNA probes were used in an *in situ* RNA–RNA hybridization to relate the 16S sequence to the epitheliocystis present on the gills of the salmon. In this study, we provide additional sequence information about the rRNA genes, including 23S, and several core genes to provide a basis for a precise taxonomic affiliation of this *Candidatus* species according to the recommendations given by several students of bacteria and *Chlamydiae* taxonomy (Everett et al. 1999; Stackebrandt 2009; Greub 2013; Everett 2014; Kim et al. 2014; Pillonel et al. 2015). The rRNA genes of members of *Simkaniaceae* have 16S and 23S sequences that are more than 90% identical to those of *Simkania negevensis* (Everett 2014). Unlike all other *Chlamydiae* some of the *Simkaniaceae* species have a group I intron in the I-*Cpa*I target site of the 23S rRNA gene. However, this intron is not present in the 23S rRNA gene of *Cand*. S. salmonis co-cultured in *P. perurans*. The 16S from *Cand*. S. salmonis show > 92.5% similarity to the 16S from *S. negevensis* suggesting that it belongs to the family Simkaniaceae (Nylund et al. 2015). The 16S from the cultured *Cand*. S. salmonis is slightly different from that described from epitheliocystis of the gill of Atlantic salmon (99.2% identity), but represents the same species according to requirement of 98.65% identity for members of the same species (Kim et al. 2014). The 23S of the cultured *Cand*. S. salmonis showed 88.1% similarity to *S. negevensis* which according to the classification scheme suggested by Pillonel et al. (2015) would suggest that it belongs to a different family. However, other members of family *Simkaniaceae*, such as *Cand*. Fritschea bemisiae (accession no: AY140910), do also show < 91.0% identity in the 23S sequence suggesting that this criterion should not be seen as absolute.

The sequence identities of FtsK, PepF, Adk, and HemL (71.1, 63.6, 69.3 and 62.4%) all show that *Cand*. S. salmonis is not the same species as *S. negevensis*, according to Pillonel et al. (2015).

The putative protein sequence identities of DnaA, SucA and FabI from *Cand*. S. salmonis compared to *S. negevensis* are 71.8, 64.6 and 71.8%, respectively. Following the classification scheme of Pillonel et al. (2015), this would imply that they belong to the same genus although one gene (Hyp325) could not be identified in our study and may have suggested placement in a different genus. Pillonel et al. (2015) suggested that in case of conflicting results a majority rule should be considered. However, even if the missing Hyp325 should give a conflicting result compared to the other three protein sequences *Cand*. S. salmonis would still be classified as a member in genus *Simkania* according to this scheme. The phylogeny based on the 16S rRNA shows a clear distinction between the genera Syngnamydia and *Simkania*, with members of genus *Fritschea* as the closest relatives to *Cand*. Syngnamydia. There is also a higher 23S rRNA sequence identity between members of *Fritschea* and *Cand*. Syngnamydia compared to the identity between *Simkania* and *Cand*. Syngnamydia. The group I intron found in the 23S rRNA gene in *Simkania* (Everett 2014) is not present in *Cand*. S. salmonis cultured in *P. perurans*. Taking into consideration the low identity between the rRNA genes of *Simkania* and *Cand*. S. salmonis, and that the sequence identities of DnaA, SucA and FabI from *Cand. S. salmonis* compared to that of *S. negevensis*, are only marginally above the proposed thresholds, we suggest that *Cand*. S. salmonis should still be placed in the *Candidatus* genus Syngnamydia.

The minimal standards for description of new species within the order Chlamydiales are not fulfilled for *Cand*. S. salmonis. However, the growth of *Cand*. S. salmonis in co-culture with *P. perurans* provides a culture system for this candidatus species and together with the genetic information provided in this study a good starting point for future description of *Cand*. S. salmonis as full species has been established. This is the first epitheliocystis agent from fish that has been cultured and genotyped according to the recommendations given by Pillonel et al. (2015).

The rRNA genes and the sequences of DnaA, SucA, FabI, FtsK, PepF, Adk, and HemL from *Candidatus* Syngnamydia salmonis cultured in *P. perurans* have been deposited in the GenBank with accession numbers: KY427067, KY427068, and MG017618–MG017635.
